# Evaluation of artificial intelligence in the therapy of oropharyngeal squamous cell carcinoma: De-escalation via Claude 3 Opus, Vertex AI and ChatGPT 4.0? – an experimental study

**DOI:** 10.1097/JS9.0000000000002139

**Published:** 2024-11-22

**Authors:** Benedikt Schmidl, Tobias Hütten, Steffi Pigorsch, Fabian Stögbauer, Cosima C. Hoch, Timon Hussain, Barbara Wollenberg, Markus Wirth

**Affiliations:** a Department of Otolaryngology Head and Neck Surgery, Technical University Munich, Munich, Germany; b Department of Radio Oncology, Technical University Munich, Munich, Germany; c Technical University Munich, Institute of Pathology, Munich, Germany

## Introduction

Highlights

- Utilizing AI in the form of novel advanced LLMs in the MDT setting of oropharyngeal squamous cell carcinoma (OPSCC) results in different treatment strategies with significant differences between HPV-positive and negative OPSCC. AI is, therefore, able to assist surgical and oncological decision-making for OPSCC and potentially other cancer types and treatment settings.
- Patient stratification with advanced LLMs is possible since the implications of HPV and the potential of therapy de-escalation are known concepts compared to prior LLMs.
- There are still limitations of the current landscape of AI tools that require further validation through clinical trials to confirm their effectiveness in surgical decision-making.

Oropharyngeal squamous cell carcinoma (OPSCC) is a heterogeneous disease with a rising incidence of human papillomavirus virus (HPV)-associated OPSCC in recent decades. These are treated by surgery or primary radio(chemo)therapy and demonstrate better survival rates compared with HPV-negative cases, leading to investigations into different treatment and de-escalation strategies^1^.

The complexity of the decision-making process is the reason that the treatment of OPSCC is currently discussed in multidisciplinary tumor boards (MDT)^2^. Artificial intelligence (AI) has opened ways to use large language models (LLMs) for the MDT, which are able to analyze and summarize large datasets of the most recent clinical studies, basic science, and the patient’s electronic health record. New advanced LLMs were introduced in the recent months, including Claude 3 Opus and Vertex AI, which outperform ChatGPT 4.0 in many benchmarks for AI systems^3–5^, promising to enhance the efficiency of surgical and oncological decision-making in the MDT for OPSCC.

The primary objective of this study was a comprehensive assessment of the novel tools Claude 3 Opus, ChatGPT 4.0, and Vertex AI and to investigate the current landscape of artificial intelligence to set the groundwork for future research into AI applications in surgical oncology, including OPSCC and the implications of HPV for a potential role in the stratification of patients for HPV de-escalation and assistance in the MDT.

## Materials and Methods

**Figure 1:**
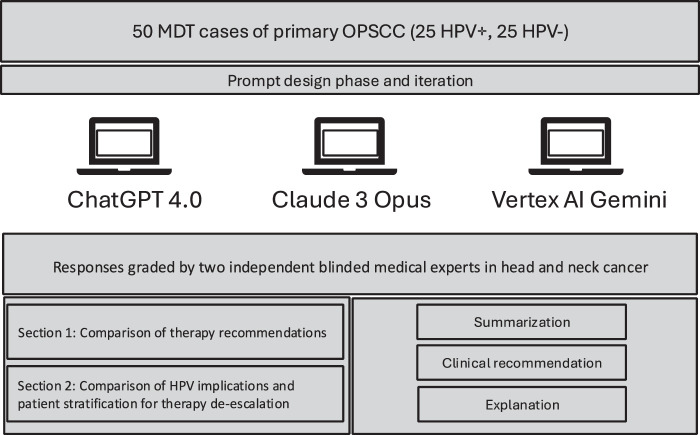
Flowchart of the overall study design. Depiction of the grading of responses by ChatGPT 4.0, Claude 3 Opus, and Vertex AI. Evaluation of the responses by two independent reviewers and in the categories of summarization, clinical recommendation, and explanation.

Fifty consecutive HPV-positive and HPV-negative cases without evidence of distant metastasis were included in this study and the electronic patient file and MDT documents provided clinical and histological tumor characteristics. The study design is depicted in Figure 1. This study was approved by the ethics committee (2024-184_1-S-NP).

For the LLMs Claude 3, ChatGPT 4.0, and Vertex AI various prompts were initially tested, and a standardized prompt format was employed to input patient information. The prompt was as follows: ‘A (XX) year old patient with a cT (XX) cN (XX) squamous cell carcinoma of the (XX), the patient (XX) smokes, (XX) and has the following secondary diseases (XX) with a Karnofsky Index of (XX). The patient is presented in an interdisciplinary tumor board. What treatment options are available and which option do you think leads to the best prognosis?’. The next question was ‘What are the implications of p16/HPV?’ and whether de-escalation therapy is an option.

Two independent blinded reviewers evaluated the responses in terms of recommendations with objective and subjective criteria and the grades of summarization, clinical recommendation, and explanation^6^. Cohen’s kappa coefficient measured inter-rater reliability and the Mann–Whitney *U* test was used to identify significant differences between the LLMs.

## Results

**Figure 2:**
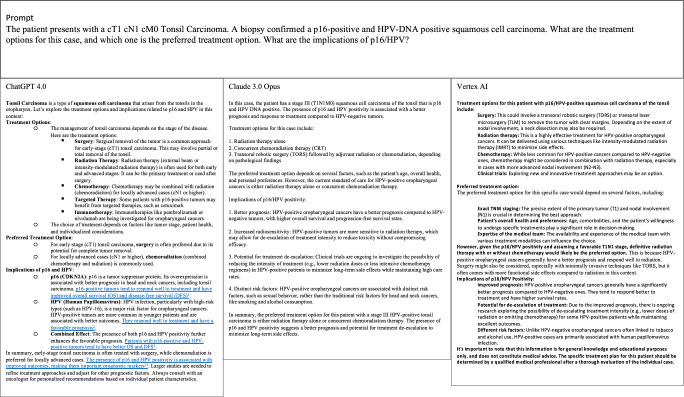
Depiction of an exemplary HPV-positive OPSCC case and the resulting response by ChatGPT 4.0, Vertex AI, and Claude 3 Opus. The prompt and the corresponding responses are shown. The answers were copied from the response page.

**Figure 3:**
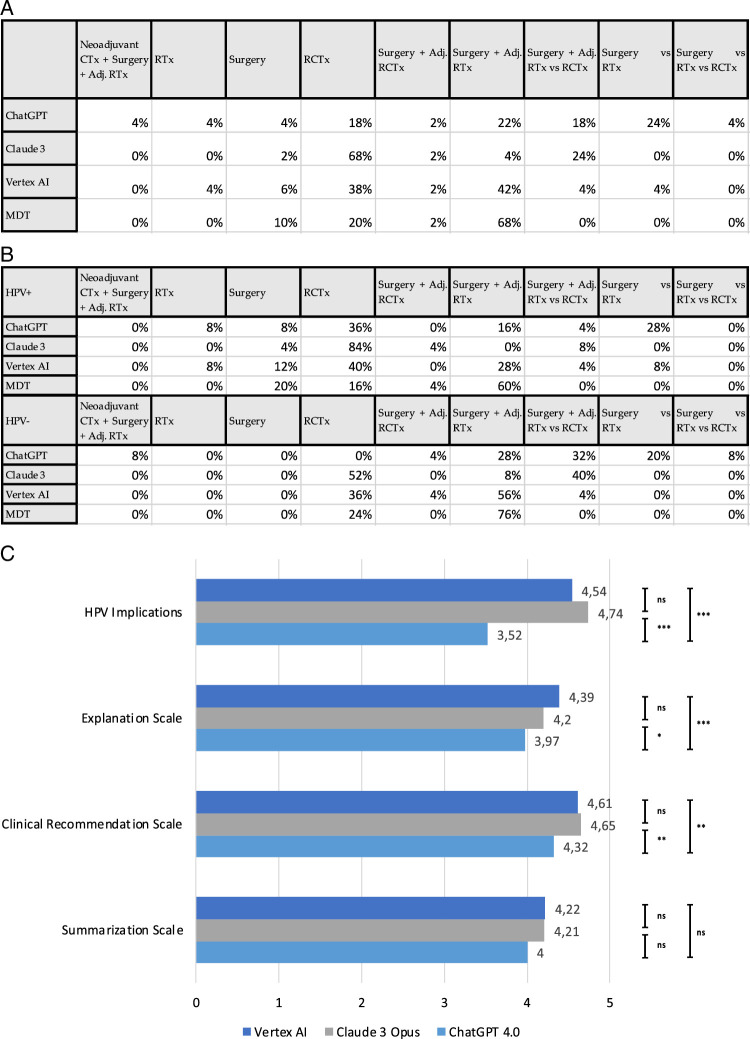
Evaluation of the performance of Vertex AI, Claude 3 Opus, and ChatGPT 4.0. (A) Comparison of the therapy recommendations by ChatGPT 4.0, Claude 3 Opus, and Vertex AI. In total nine different treatment strategies were recommended. Depicted is the percentage out of 50 cases. (B) Subgroup analysis of the therapy recommendations based on the p16/HPV status of the OPSCC. Depicted is the percentage out of 25 cases for HPV+ OPSCC and 25 cases for HPV- OPSCC. (C) Comparison of the grading of summarization of text, clinical recommendation, explanation, and HPV implications by two independent reviewers for each of the LLMs. Each bar is the average of the two independent reviewers’ grading. ^#^number, *P>0.05, **P>0.01, ***P>0.001.

The therapy recommendations in this study involved different treatment strategies and patient stratification, with all LLMs recommending either surgery with adjuvant radiotherapy or primary radio(chemo)therapy for OPSCC. Exemplary responses are depicted in Figure 2. All three LLMs were able to correctly name the surgical concepts and indications of transoral laser surgery (TOLS) and transoral robotic surgery (TORS). Claude 3 recommended radiochemotherapy for 68% of patients, while surgery with adjuvant radiotherapy was recommended for only a few patients. Vertex AI recommended radiochemotherapy for 38% and surgery with adjuvant radiotherapy for 42% of patients (Fig. 3A). ChatGPT recommended surgery and adjuvant radiotherapy for most of the HPV-negative OPSCC and stated that surgery and radiation therapy alone are equivalent in terms of oncological outcome.

For the implications of p16/HPV, Vertex AI, and Claude 3 highlighted the improved prognosis and the potential of therapy de-escalation, and even possible biological and mechanistic explanations for HPV-discordance. ChatGPT only addressed the better prognosis but did not mention the potential risk stratification for therapy de-escalation. HPV influenced the therapy recommendations for the new LLMs, with, for example, Claude 3 recommending radio(chemo)therapy for 84% of HPV-positive OPSCC and only 52% of HPV-negative OPSCC. Vertex AI recommended surgery and adjuvant radiotherapy for the majority of HPV-negative patients (56%), and radiochemotherapy for HPV-positive OPSCC (40%). The actual MDT recommended surgery and adjuvant radiotherapy for 60% of HPV-negative and 76% of HPV-negative cases (Fig. 3A, B).

The three LLMs reached similar scores for clinical recommendation explanation and summarization, with ChatGPT 4.0 consistently ranked the lowest in comparison, even in the category of HPV implications. For explanation and clinical recommendation, ChatGPT 4.0 was significantly less performant than Vertex AI and Claude 3, whereas, in terms of summarization, both LLMs performed slightly better than ChatGPT 4.0 without a statistically significant difference (Fig. 3C). The agreement of the two reviewers ranged from substantial to fair (Supplementary Material).

The LLMs highlighted that the final decision needs to be discussed with a physician after careful consideration of the patient’s preferences, overall health status, and potential risks and benefits of each approach and also addressed their own limitations.

## Discussion

This is the first study comparing the three advanced LLMs, Vertex AI, Claude 3 Opus, and ChatGPT 4.0 for evaluating patient stratification for HPV de-escalation for 50 primary OPSCC.

Vertex AI and Claude 3 Opus showed significantly superior performance in terms of clinical recommendation and explanation for both HPV-positive and HPV-negative cases and potential for patient stratification and understanding of HPV implications. At the same time the limitations of the current landscape of LLMs were highlighted in this study with variations in regional treatment guidelines, especially in the form of HPV de-escalation strategies^7,8^, a lack of transparency of AI called black box phenomenon^9^, the importance of accurate prompt design^2,10^, and the inability to tailor individual patient treatment plans. Currently even the most advanced LLMs are restrained to being an auxiliary tool rather than a replacement of the MDT and need to be evaluated carefully by medical professionals.

## Conclusions

This study represents the first comparison of the performance of the advanced LLMs, Vertex AI, and Claude 3 Opus, in evaluating primary cases of OPSCC for HPV de-escalation strategies. Claude 3 and Vertex AI proved to be superior and demonstrated impressive knowledge about the implications of HPV for the therapy and characteristics of OPSCC, laying the groundwork for further research into the integration of novel AI tools into surgical and oncological decision-making and pave the way for further research in other cancer types and treatment settings.

## Ethical approval

The study was conducted according to the guidelines of the Declaration of Helsinki and the ethics committee of the Technical University of Munich (Reference: 2024-184_1-S-NP).

## Consent

Patients consent was waived by the ethics committee of the Technical University of Munich due to the anonymized data that was used in this study.

## Source of funding

This research received no external funding or sponsorship.

## Author contribution

B.S. and M.W.: conceptualization; B.S., T.H., M.W., B.W., S.P., C.C.H., and T.H.: formal analysis; B.S. and B.W.: methodology, B.W.: validation; B.S., M.W., T.H., C.C.H., F.S., and S.P.: writing – review and editing. All authors have read and agreed to the published version of the manuscript.

## Conflicts of interest disclosure

The authors declare no conflicts of interest.

## Research registration unique identifying number (UIN)

1. Name of the registry: German Clinical Trials.
2. Unique identifying number or registration ID: DRKS00034390.
3. Hyperlink to your specific registration (must be publicly accessible and will be checked): https://drks.de/search/de/trial/DRKS00034390

## Guarantor

B. Schmidl.

## Data availability statement

The generated datasets of this study are in the supplementary material.

## Provenance and peer review

This paper was not invited.
